# Requirements for the import of neisserial Omp85 into the outer membrane of human mitochondria

**DOI:** 10.1042/BSR20130007

**Published:** 2013-03-13

**Authors:** Christine Ott, Mandy Utech, Monika Goetz, Thomas Rudel, Vera Kozjak-Pavlovic

**Affiliations:** Department of Microbiology, Biocentre, University of Würzburg, 97074 Würzburg, Germany

**Keywords:** β-barrel, mitochondrion, Omp85, PorB, POTRA domain, BAM, β-barrel assembly machinery, BN, blue native, HEK-293T, human embryonic kidney 293T, IMM, inner mitochondrial membrane, IMS, intermembrane space, OMM, outer mitochondrial membrane, PEI, polyethylenimine, POTRA, polypeptide-transport associated, SAM, sorting and assembly machinery, SDHA, succinate dehydrogenase A, TOM, translocase of the outer mitochondrial membrane

## Abstract

β-Barrel proteins are present only in the outer membranes of Gram-negative bacteria, chloroplasts and mitochondria. Fungal mitochondria were shown to readily import and assemble bacterial β-barrel proteins, but human mitochondria exhibit certain selectivity. Whereas enterobacterial β-barrel proteins are not imported, neisserial ones are. Of those, solely neisserial Omp85 is integrated into the outer membrane of mitochondria. In this study, we wanted to identify the signal that targets neisserial β-barrel proteins to mitochondria. We exchanged parts of neisserial Omp85 and PorB with their *Escherichia coli* homologues BamA and OmpC. For PorB, we could show that its C-terminal quarter can direct OmpC to mitochondria. In the case of Omp85, we could identify several amino acids of the C-terminal β-sorting signal as crucial for mitochondrial targeting. Additionally, we found that at least two POTRA (polypeptide-transport associated) domains and not only the β-sorting signal of Omp85 are needed for its membrane integration and function in human mitochondria. We conclude that the signal that directs neisserial β-barrel proteins to mitochondria is not conserved between these proteins. Furthermore, a linear mitochondrial targeting signal probably does not exist. It is possible that the secondary structure of β-barrel proteins plays a role in directing these proteins to mitochondria.

## INTRODUCTION

β-Barrel proteins can be found in the outer membranes of Gram-negative bacteria, chloroplasts and mitochondria. There they form pores that can have various functions, but essentially enable communication between different compartments. In case of bacteria, these compartments are the periplasmic space and the environment. In the case of the two organelles, those are the IMS (intermembrane space) and the cytosol [1].

In bacteria, approximately 2–3% of genes encode β-barrel proteins [1]. Bacterial β-barrel proteins are produced in the cytosol and directed to the Sec machinery by a signal peptide. The Sec machinery transports them into the periplasmic space where the targeting sequence is removed by the signal peptidase. Subsequently, bacterial β-barrel proteins are inserted into the bacterial outer membrane by the BAM (β-barrel assembly machinery) [2]. The central component of the BAM is BamA, also known as Omp85, first identified in *Neisseria meningitidis* [3]. BamA/Omp85 is accompanied by several other accessory lipoproteins required for the assembly of bacterial β-barrel proteins, but their number and significance vary among different bacteria [2,4]. BamA/Omp85 consists of a membrane β-barrel domain and of a periplasmic part containing five POTRA (polypeptide-transport associated) domains [5]. Functioning of BamA/Omp85 in the assembly of β-barrel proteins was reported to depend only on the last one of the POTRA domains [6]. Membrane integration and assembly of bacterial β-barrel proteins depends on a sorting signal present at the very end of the protein. In particular the last carboxy (C)-terminal amino acid is important–hydrophobicity and the aromatic nature of the amino acid are crucial, and in most cases the last C-terminal amino acid is phenylalanine [5,7].

The assembly of β-barrel proteins is evolutionary conserved and follows a similar route in mitochondria. Mitochondrial β-barrel proteins are produced on cytosolic ribosomes and imported into the IMS by the TOM [translocase of the OMM (outer mitochondrial membrane)] complex. From the IMS side, mitochondrial β-barrel proteins are recognized by the SAM (sorting and assembly machinery), also known as the TOB (topogenesis of β-barrel proteins) complex [8,9], and integrated into the OMM. The central component of the complex, Sam50/Tob55, exhibits sequence similarity with BamA/Omp85, but contains only one POTRA domain [10]. The β-sorting signal that directs mitochondrial β-barrel proteins to the SAM complex also appears similar to the one present in bacterial β-barrel proteins [11]. However, the presence of the C-terminal phenylalanine is not of crucial importance for the assembly of β-barrel proteins in mitochondria. Also, whereas in mammalian cells the β-sorting signal has to be located at the very end of a β-barrel protein to be recognized, in yeast cells this is not an absolute requirement [11,12].

It has been proposed by several experiments performed with fungal mitochondria that the biogenesis of β-barrel proteins is evolutionary conserved in such a way that mitochondria will recognize and import bacterial β-barrel precursors, as well as integrate them into the OMM [13]. Likewise, it seems that mitochondrial β-barrel proteins can be recognized by the BAM complex and integrated into the bacterial outer membrane [14]. Our data from experiments with human cells showed, however, that human mitochondria readily import only β-barrel proteins from *Neisseria* spp., whereas those from enterobacteria are not recognized. In addition, neisserial Omp85 proteins are integrated into the OMM with the help of the SAM complex, but neisserial PorB proteins, although targeted to mitochondria, are not recognized by the SAM complex and do not form OMM complexes. However, mitochondria-localized neisserial Omp85 was able to insert PorB molecules into the OMM, showing that Omp85 is capable of functioning in a lipid bilayer without any accessory lipoproteins [15].

Considering the high sequence and structural similarity between neisserial Omp85 and enterobacterial BamA proteins, we wondered why the former is taken up by human mitochondria, whereas the latter is not. We exchanged parts of these two proteins to identify a C-terminal domain of Omp85 as important for its mitochondrial targeting. Shortening and mutation of several of the last C-terminal amino acids of Omp85 identified the last phenylalanine and the glutamine at position 787 as crucial for directing this protein to mitochondria. We also explored the role of the POTRA domains in the assembly and functioning of Omp85 in the OMM. We could show that POTRA4 and 5 are crucial for the OMM integration of Omp85 and for the functioning of this protein in the OMM assembly of its substrate PorB. Furthermore, we could demonstrate that the C-terminus is important for mitochondrial targeting of PorB, another neisserial β-barrel protein, but we could not identify any conserved targeting sequence. Our results support the idea that secondary structures, rather than linear signals, play a role in the targeting and OMM integration of β-barrel proteins.

## EXPERIMENTAL

### Cell culture and transfection

HeLa cells and HEK-293T (human embryonic kidney 293T) cells were cultivated in RPMI 1640 (Gibco) and DMEM (Dulbecco's modified Eagle's medium; Gibco) medium supplemented with 10% (v/v) FBS (fetal bovine serum; Biochrom) and 1% (v/v) penicillin/streptomycin (Invitrogen). HeLa cells were transfected using either Lipofectamine™ 2000 (Invitrogen) according to the manufacturer's protocol or PEI (polyethylenimine) (Polysciences). For PEI transfection, 8 μl of PEI was mixed with 2 μg of plasmid DNA and 100 μl of OPTIMEM. After 10 min of incubation at room temperature, the mixture was added to cells seeded on coverslips in a 12-well plate containing 400 μl of fresh medium. The medium was exchanged 6–8 h post-transfection. HEK-293T cells were transfected using calcium phosphate precipitation as described previously [15].

### Microscopy

Immunofluorescence microscopy was performed as described previously [16].

### Biochemical methods

Genes for the proteins used in this study were obtained by PCR from the plasmids FLAG-PorB*_Ngo_* [16], FL-Omp85*_Ngo_*, FLAG-OmpC and FLAG-BamA [17]. Proteins were cloned into the pcDNA3 vector (Invitrogen) with an amino-terminal FLAG-tag.

Isolation of the crude mitochondrial fraction, BN (blue native)-PAGE and Western blot were performed as described before [18,19]. Samples for BN-PAGE analysis were solubilized in 1% (w/v) digitonin buffer [1% (w/v) digitonin (Sigma) in 20 mM Tris/HCl, 0.1 mM EDTA, 1 mM PMSF, 50 mM NaCl, 10% (v/v) glycerol, pH 7.4]. For sequence alignment of the proteins, the ClustalW2 programme was used.

### Antibodies

FLAG antibody was purchased from Sigma, Tom40 antibody from Santa Cruz Biotechnology, Tom20 from BD Biosciences and SDHA (succinate dehydrogenase A) from Invitrogen. Fluorochrome-coupled secondary antibodies used for immunofluorescence microscopy were purchased from Jackson Immuno Research.

## RESULTS

### C-terminal part of Omp85*_Ngo_* is important for its mitochondrial localization

**Figure 1:**
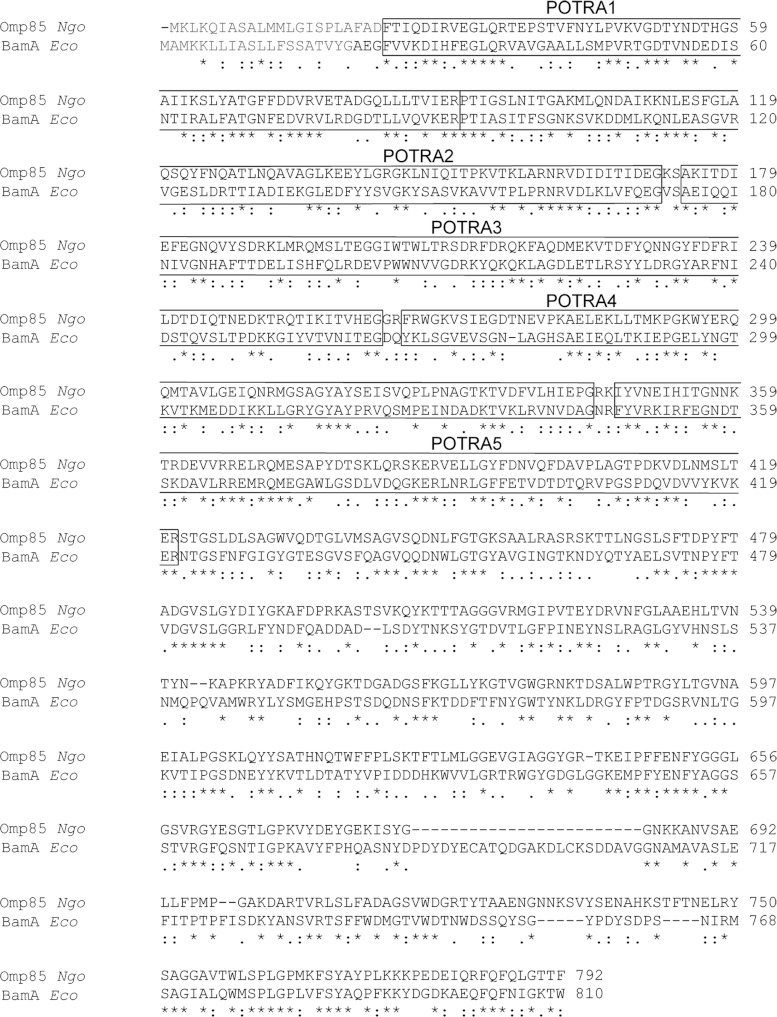
Omp85*_Ngo_* and BamA*_Eco_* are homologous proteins that possess five POTRA domains The amino acid sequences of Omp85*_Ngo_* and BamA*_Eco_* were aligned using ClustalW2. The five predicted POTRA domains of the proteins are marked with a box. Light grey residues represent the bacterial signal sequence.

The OMM proteins Omp85 of *N. gonorrhoeae* (*Ngo*) and BamA of *Escherichia coli* (*Eco*) are homologous proteins. Both possess five POTRA domains followed by a transmembrane domain (Figure 1) [20,21]. Recently, we could show that only Omp85*_Ngo_* but not BamA*_Eco_* is imported into human mitochondria and assembled in the OMM [15]. Therefore we were presented with two similar proteins that exhibited a difference regarding their targeting to mitochondria.

**Figure 2:**
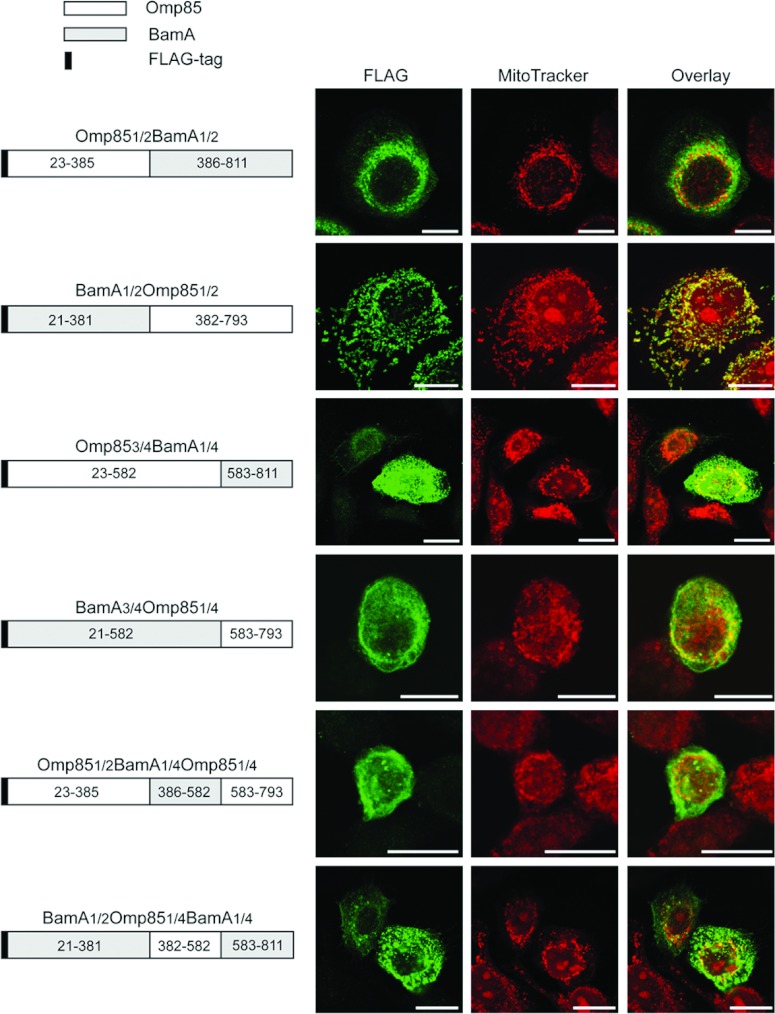
Rear half of Omp85*_Ngo_* is required for mitochondrial targeting but the last quarter of the protein is not sufficient to mediate import The scheme on the left-hand side shows the protein part exchanges performed between Omp85*_Ngo_* and BamA*_Eco_*; indicated in the boxes are the numbers of the amino acid residues of each protein present in the chimeric constructs Omp85_1/2_BamA_1/2_, BamA_1/2_Omp85_1/2_, Omp85_3/4_BamA_1/4_, BamA_3/4_Omp85_1/4_, Omp85_1/2_BamA_1/4_Omp85_1/4_ and BamA_1/2_Omp85_1/4_BamA_1/4_. All constructs have an N-terminal FLAG-tag and do not contain the bacterial signal sequence. HeLa cells were transfected with these plasmids, labelled using the Δψ-sensitive dye MitoTracker (red) and an antibody against the FLAG-tag (green) and analysed by fluorescence microscopy. Scale bars represent 100 μm.

In order to elucidate which part of Omp85*_Ngo_* is required for mitochondrial targeting, we created the chimeric proteins Omp85_1/2_BamA_1/2_ and BamA_1/2_Omp85_1/2_, consisting of the first half of Omp85*_Ngo_* and the second of BamA*_Eco_* and vice versa. The genes for these chimeras were cloned into a pcDNA3-based vector, introducing an amino (N)-terminal FLAG-tag (Figure 2). HeLa cells were transfected with the constructs, decorated with the membrane potential (Δψ)-sensitive dye MitoTracker and an antibody against the FLAG-tag and analysed by fluorescence microscopy. We observed that only the BamA_1/2_Omp85_1/2_ chimerical protein containing the C-terminal half of Omp85*_Ngo_* co-localized with mitochondria. Mitochondria retained their membrane potential indicating, as discussed before [12,15], that the protein was correctly assembled in the OMM (Figure 2). To further locate the mitochondrial import sequence of Omp85*_Ngo_*, we next exchanged the C-terminal quarters of Omp85*_Ngo_* and BamA*_Eco_* and of the chimeric constructs with each other, creating the following constructs: Omp85_3/4_BamA_1/4_, BamA_3/4_Omp85_1/4_, Omp85_1/2_BamA_1/4_Omp85_1/4_ and BamA_1/2_Omp85_1/4_BamA_1/4_ (Figure 2). However, expression of these constructs in HeLa cells followed by immunofluorescence showed that none of the proteins localized to mitochondria, even when three quarters of the protein consisted of Omp85*_Ngo_* (Figure 2). We conclude that the C-terminal half, but not the C-terminal quarter of Omp85*_Ngo_* alone, is sufficient for directing the protein to mitochondria.

### β-Sorting signal of Omp85*_Ngo_* plays a role in mitochondrial targeting

**Figure 3:**
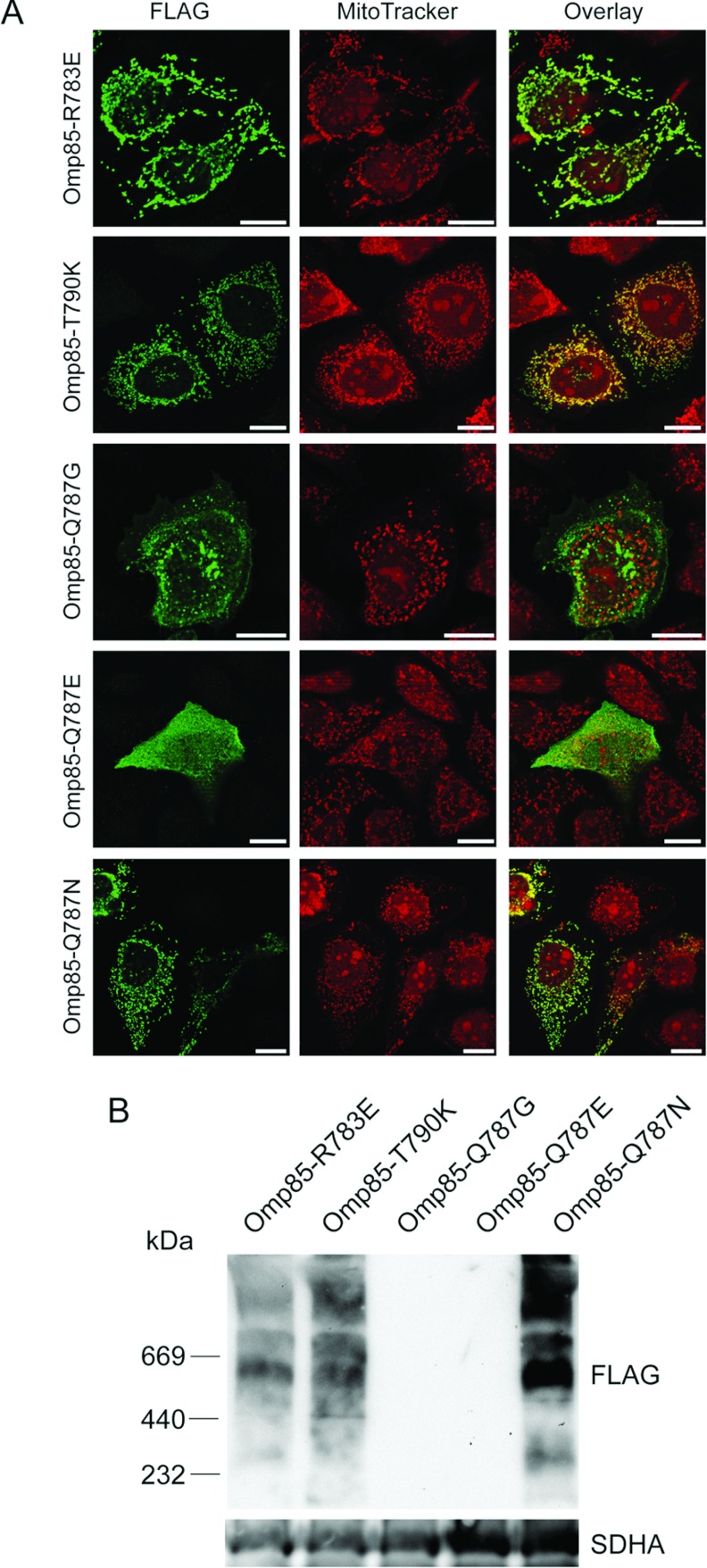
Mutation of single amino acids in the β-sorting signal of Omp85*_Ngo_* can interfere with mitochondrial targeting (**A**) HeLa cells were transfected with plasmids coding for Omp85-R783E, Omp85-T790, Omp85-Q787G, Omp85-Q787E and Omp85-Q787N, which contained a FLAG-tag at the N-terminus. The cells were stained with the Δψ-sensitive dye MitoTracker (red) followed by immunolabelling using antibodies against the FLAG-tag (green), and analysed by fluorescence microscopy. Scale bars represent 100 μm. (**B**) Mitochondria were isolated from HEK-293T cells expressing the plasmids from (A). 50 μg of mitochondrial protein was solubilized in 1% digitonin buffer and analysed by BN-PAGE and Western blot using antibodies against the FLAG-tag and SDHA as a control.

We recently demonstrated that removal of the last 12 amino acid residues of Omp85*_Ngo_*, which constitute the β-sorting signal mediating outer membrane integration, impairs mitochondrial import [15]. To further address the question of the importance of the β-sorting signal in the mitochondrial targeting of Omp85*_Ngo_*, we mutated the amino acids R783, Q787 and T790. These amino acids are part of the Omp85*_Ngo_* β-sorting signal, but are not present at the same positions in BamA*_Eco_*. The mutations R783E and T790K had no impact on the localization of Omp85*_Ngo_*, as assessed by fluorescence microscopy. On the other hand, the construct Omp85-Q787G, where a polar glutamine was exchanged with glycine, did not co-localize with mitochondria anymore (Figure 3A). To test the importance of the polarity of the glutamine residue for mitochondrial targeting of Omp85*_Ngo_*, we created the constructs Omp85-Q787N, which should behave as the wild-type, and Omp85-Q787E, where the polar glutamine is substituted by the charged glutamic acid residue. The constructs were expressed in HeLa cells and subsequent fluorescence microscopy revealed that Omp85-Q787N co-localized with mitochondria, whereas Omp85-Q787E did not (Figure 3A). Mitochondria-targeted Omp85*_Ngo_* forms complexes, most likely oligomers, in the OMM [15], so we next tested the ability of the mutant constructs to oligomerize. We expressed the constructs in HEK-293T cells, isolated mitochondria and analysed them by BN-PAGE and Western blot. In agreement to microscopy data, we observed that the protein constructs Omp85-Q787G and Omp85-Q787E, which were not targeted to mitochondria, also failed to form detectable protein complexes (Figure 3B). We conclude that the polar property of Q787 is important for mitochondrial targeting of Omp85*_Ngo_*.

**Figure 4:**
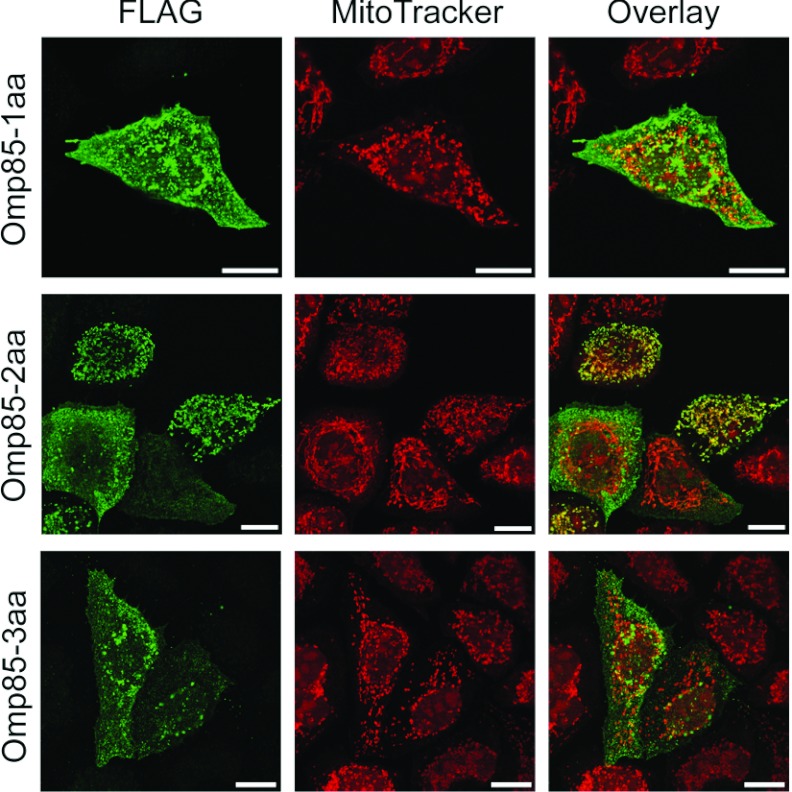
C-terminal phenylalanine plays a crucial role in mitochondrial targeting of Omp85*_Ngo_* HeLa cells were transfected with plasmids coding for the N-terminally FLAG-tagged proteins Omp85-1aa, Omp85-2aa and Omp83-3aa, where the last, two last and three last amino acids have been removed, respectively. Cells were stained using the ΔΨ-sensitive dye MitoTracker (red) and an antibody against the FLAG-tag (green) and analysed by fluorescence microscopy. Scale bars represent 100 μm.

To further explore the role of the C-terminus of Omp85*_Ngo_* in mitochondrial targeting, we shortened the protein by one to six C-terminal amino acids. It is known that in bacteria the last phenylalanine plays a crucial role in the membrane integration of β-barrel proteins [7], one of which is Omp85*_Ngo_*. When we removed the C-terminal phenylalanine of Omp85*_Ngo_*, this construct, missing only one amino acid, was not imported into mitochondria, but accumulated in the cytosol (Figure 4). Interestingly, the removal of two C-terminal amino acids, phenylalanine and threonine, led to a partial recovery of mitochondrial targeting and we observed that an estimated 40% of cells contained this construct in mitochondria. Further removal of three or more C-terminal amino acids completely prevented mitochondrial import of such shortened Omp85*_Ngo_* constructs (Figure 4). We conclude that the C-terminal phenylalanine plays an important role in the targeting of Omp85*_Ngo_* to mitochondria.

### POTRA domains are important for membrane integration and function of Omp85*_Ngo_* in mitochondria

**Figure 5:**
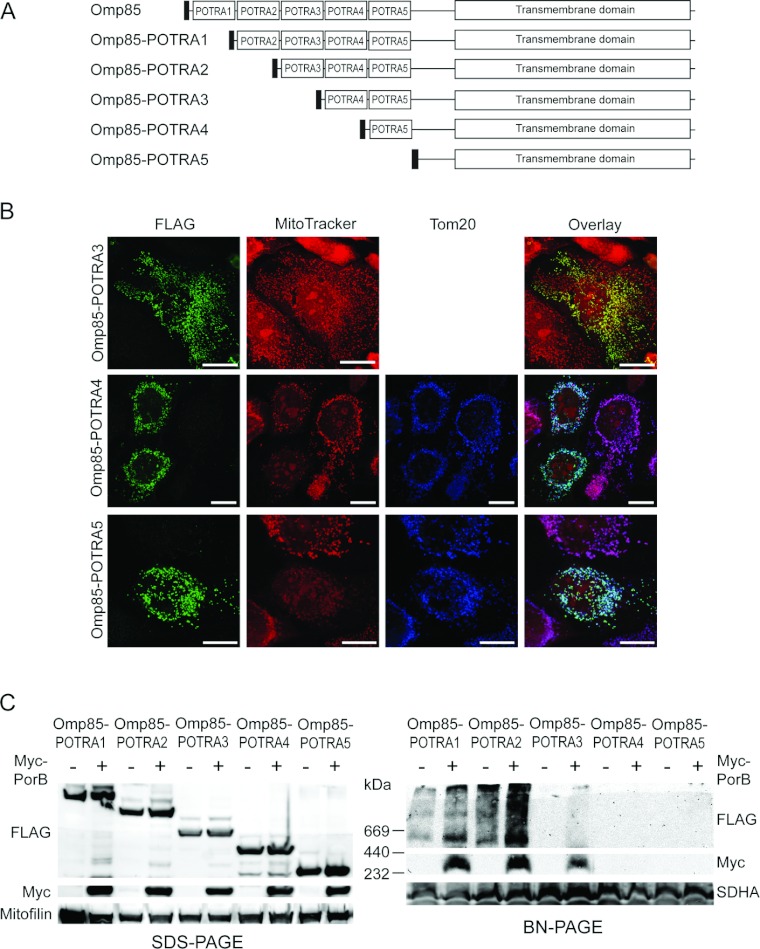
Two POTRA domains are required for Omp85*_Ngo_* OMM integration and function (**A**) Scheme of the constructed Omp85*_Ngo_* mutants lacking one to five POTRA domains. (**B**) Expression of the constructs in HeLa cells and fluorescence microscopy. Plasmids coding for the constructs Omp85-POTRA3, Omp85-POTRA4 and Omp85-POTRA5, tagged with the FLAG-tag at the N-terminus, were transfected into HeLa cells. The cells were stained with the Δψ-sensitive dye MitoTracker (red), fixed, and immunofluorescence was performed using antibodies against the FLAG-tag (green) and Tom20 (blue), followed by fluorescence microscopy. Scale bars represent 100 μm. (**C**) FLAG-tagged Omp85-POTRA1, Omp85-POTRA2, Omp85-POTRA3, Omp85-POTRA4 and Omp85-POTRA5 were expressed in HEK-293T cells without or together with PorB containing a Myc-tag at the N-terminus. Mitochondria were isolated and 50 μg of mitochondrial protein was analysed by SDS/PAGE and Western blot using antibodies against the FLAG-tag, the Myc-tag and Mitofilin as a control, and by BN-PAGE followed by Western blotting using antibodies against the FLAG-tag, the Myc-tag and SDHA as a control.

A study by Bos et al. [6] showed that Omp85 of *N. meningitidis* requires only a single POTRA domain to be functional and mediate integration of OMM proteins [6]. In order to test whether the same applies to the assembly and function of Omp85*_Ngo_* in human mitochondria, we constructed mutants lacking one to five POTRA domains (Figure 5A). Expression of the FLAG-tagged constructs in HeLa cells showed that all still co-localized with mitochondria (Figure 5B and not shown). Expression of Omp85-POTRA4 and Omp85-POTRA5, however, caused fragmentation of mitochondria and loss of Δψ, indicating that these constructs could not be assembled in the OMM anymore and accumulated in the IMS/IMM (inner mitochondrial membrane) compartment [12].

We could show before that mitochondria-localized and correctly assembled Omp85*_Ngo_* can mediate the assembly of its natural substrate PorB*_Ngo_* in the OMM of human mitochondria [15]. In order to investigate whether the constructs lacking different numbers of POTRA domains can still exhibit this function, we expressed them in HEK-293T cells alone and together with N-terminally Myc-tagged PorB*_Ngo_* and isolated the mitochondria. Analysis of mitochondria by SDS/PAGE and Western blot showed that both proteins were present in the anticipated samples and expressed in comparable amounts (Figure 5C, left panel). We then analysed the same mitochondria with BN-PAGE followed by Western blot. We observed that up to three POTRA domains can be removed without impairing the ability of Omp85 to integrate PorB*_Ngo_* in the OMM (Figure 5C, right panel). Interestingly, Omp85-POTRA3 formed almost no complexes in the mitochondrial OMM. This suggests that Omp85 lacking three POTRA domains is present mostly in monomers but is still at least partially functional in PorB*_Ngo_* assembly. When four or all five POTRA domains of Omp85*_Ngo_* were removed, we could detect no Omp85*_Ngo_* complexes and these constructs could not assemble PorB*_Ngo_* in the OMM (Figure 5C, right panel). This confirmed the microscopy data (Figure 5B), which indicated that these two proteins probably accumulated in the IMS as inactive aggregates. Taken together, our data suggest that, in human mitochondria, Omp85*_Ngo_* requires at least two POTRA domains for the integration and proper function.

### C-terminus, but not β-sorting signal, directs PorB*_Ngo_* to mitochondria

**Figure 6:**
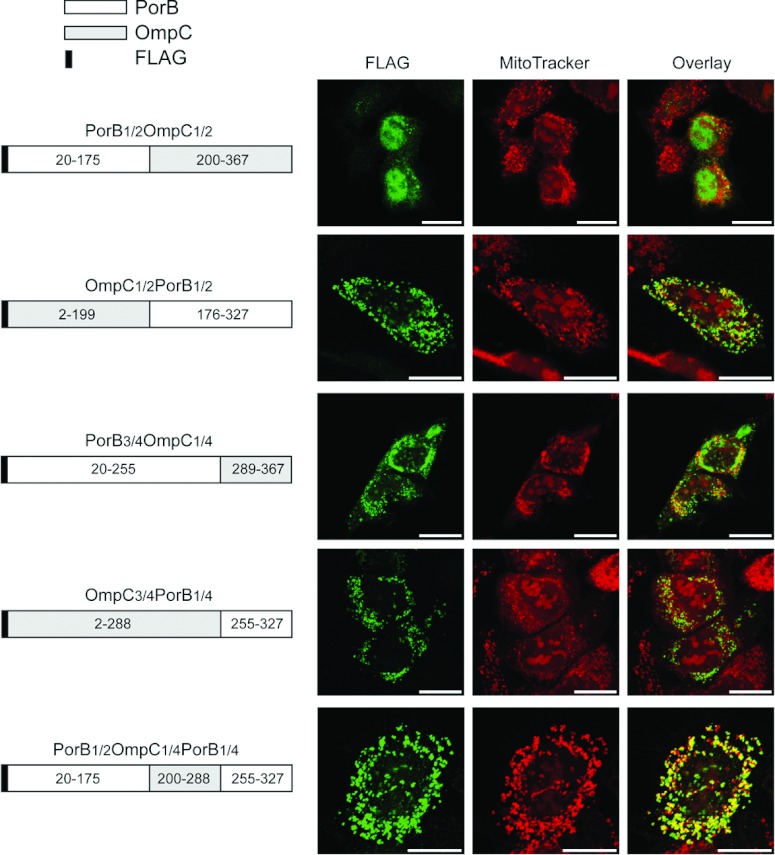
C-terminal quarter of PorB*_Ngo_* is sufficient for mitochondrial import HeLa cells were transfected with plasmids containing the information for the N-terminally FLAG-tagged proteins PorB_1/2_OmpC_1/2_, OmpC_1/2_PorB_1/2_, PorB_1/2_OmpC_1/4_PorB_1/4_, PorB_3/4_OmpC_1/4_ and OmpC_3/4_PorB_1/4_, and analysed by fluorescence microscopy using the Δψ-sensitive dye MitoTracker (red) and an antibody against the FLAG-tag (green). Scale bars represent 100 μm. The schemes of the protein part exchanges show the numbers of the amino acids of each protein present in the chimeric constructs. All constructs do not contain a bacterial signal sequence.

Similar to Omp85*_Ngo_* and BamA*_Eco_*, only neisserial PorB*_Ngo_* but not the homologous *E. coli* protein OmpC*_Eco_* is imported into human mitochondria. PorB*_Ngo_*, unlike Omp85*_Ngo_*, does not integrate into the OMM [15]. We were curious whether the results obtained from the experiments with Omp85*_Ngo_* and BamA*_Eco_* could be applied to PorB*_Ngo_* and OmpC*_Eco_* as well. We exchanged the halves of these two proteins to elucidate which part of PorB*_Ngo_* is required for mediating its import into mitochondria, and cloned the FLAG-tagged constructs PorB_1/2_OmpC_1/2_ and OmpC_1/2_PorB_1/2_. After the expression of these proteins in HeLa cells, only OmpC_1/2_ PorB_1/2_ co-localized with mitochondria, which lost the Δψ (Figure 6). Therefore similar to Omp85*_Ngo_*, the C-terminal half of PorB*_Ngo_* appears to contain the signal for mediating mitochondrial import of this β-barrel protein. We next shuffled the last quarters of PorB*_Ngo_*, OmpC*_Eco_* and PorB_1/2_-OmpC_1/2_, obtaining the following FLAG-tagged constructs: PorB_3/4_OmpC_1/4_, OmpC_3/4_PorB_1/4_ and PorB_1/2_OmpC_1/4_PorB_1/4_ (Figure 6). The proteins were expressed in HeLa cells, which were analysed by fluorescence microscopy. PorB_1/2_OmpC_1/4_PorB_1/4_ co-localized exclusively with mitochondria. OmpC_3/4_PorB_1/4_ co-localized with mitochondria in most of the cases with some protein remaining cytosolic, whereas PorB_3/4_OmpC_1/4_ mostly aggregated in the cytosol, although a portion of this protein was also imported into mitochondria (Figure 6). In all cases, mitochondria lost the Δψ upon import of the expressed protein. We conclude that the C-terminal quarter of PorB*_Ngo_* is important for its mitochondrial targeting.

**Figure 7:**
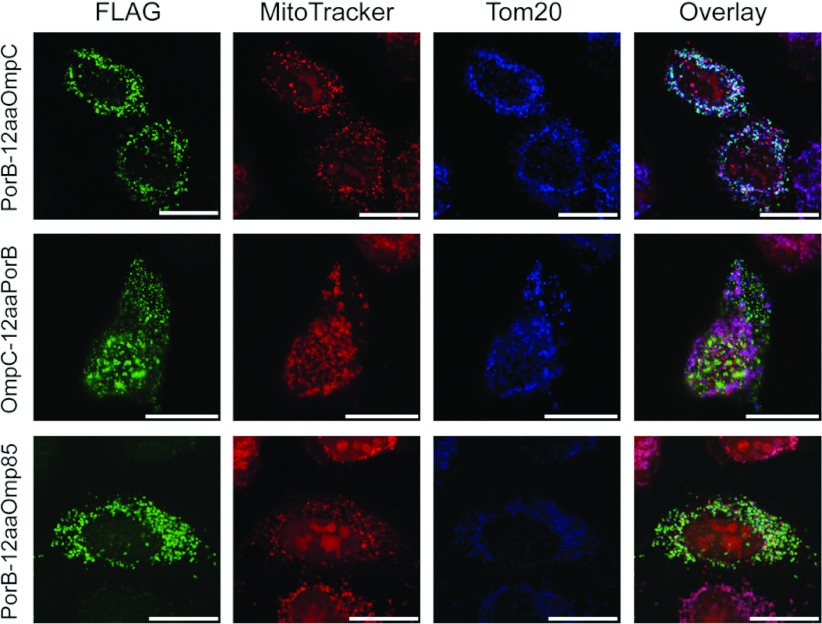
Mutations of the β-sorting signal of PorB do not prevent import and cannot mediate OMM insertion HeLa cells were transfected with plasmids coding for the N-terminally FLAG-tagged proteins PorB-12aaOmpC (the last 12 amino acids of PorB*_Ngo_* exchanged with the last 12 amino acids of OmpC*_Eco_*), OmpC-12aaPorB (the last 12 amino acids of OmpC*_Eco_* exchanged with the last 12 amino acids of PorB*_Ngo_*) and PorB-12aaOmp85 (where the last 12 amino acids of PorB*_Ngo_* were exchanged with the last 12 amino acids of Omp85*_Ngo_*). The cells were stained with the Δψ-sensitive dye MitoTracker (red), followed by immunofluorescence using antibodies against the FLAG-tag (green) and the OMM protein Tom20 (blue), and analysed by confocal microscopy. Scale bars represent 100 μm.

Since the β-barrel signal is localized in the C-terminal quarter, we wanted to know if it also plays a role in mitochondrial targeting of PorB*_Ngo_* as it does for Omp85*_Ngo_*. We therefore exchanged the last 12 amino acid residues of PorB*_Ngo_* with those of OmpC*_Eco_* and vice versa, and expressed the constructs named PorB-12aaOmpC and OmpC-12aaPorB in HeLa cells. Microscopy studies revealed that the protein PorB-12aaOmpC co-localized with mitochondria and led to the loss of Δψ similar to PorB*_Ngo_*, whereas OmpC-12aaPorB remained cytosolic (Figure 7). Therefore, in contrast to Omp85*_Ngo_*, changes in the β-sorting signal of PorB*_Ngo_* do not affect its mitochondrial targeting. Likewise, the β-sorting signal of PorB*_Ngo_* alone is not sufficient for targeting other proteins to mitochondria. Finally, we addressed the question whether substituting the β-sorting signal of PorB*_Ngo_* with the one of Omp85*_Ngo_* can mediate integration of PorB*_Ngo_* into the OMM. We created the FLAG-tagged construct PorB-12aaOmp85, where the last 12 amino acids of PorB*_Ngo_* were exchanged by the last 12 amino acids of Omp85*_Ngo_*. Microscopy studies of transfected HeLa cells showed that PorB-12aaOmp85 co-localized with mitochondria but that this led nevertheless to the loss of Δψ and mitochondrial fragmentation, indicating that the protein was not integrated into the OMM (Figure 7). Therefore the β-sorting signal of Omp85*_Ngo_* cannot mediate PorB*_Ngo_* integration into the OMM.

## DISCUSSION

Recently, we could show that human mitochondria, in contrast to their fungal counterparts, exhibit a certain specificity regarding the import and assembly of bacterial β-barrel proteins. We observed that β-barrel proteins of neisserial, but not enterobacterial origin were targeted to mitochondria. Of these, solely Omp85 proteins could integrate into the OMM, whereas PorB proteins entered mitochondria, but did not assemble in the OMM. Owing to the IMS/IMM aggregation, PorB caused mitochondrial fragmentation and loss of Δψ [15]. This indicates that there must be a crucial sequence or structure differences between neisserial β-barrel proteins and their homologues in other bacteria that are the cause of different targeting. In this study, we aimed to elucidate what these differences were and which protein parts were responsible for mediating mitochondrial import.

We first exchanged parts of Omp85*_Ngo_* with those of the homologous BamA*_Eco_*. We could establish that the C-terminal half of Omp85*_Ngo_* is required for mitochondrial targeting (Figure 2). Our further experiments showed the special role of the β-sorting signal and the last phenylalanine in the targeting of Omp85*_Ngo_* to mitochondria (Figure 4). Also, the polar property of the glutamine at position 787 seems to be of special importance, as mutating it abrogates mitochondrial targeting of Omp85*_Ngo_* (Figure 3). However, taken together, these results do not indicate a linear sequence that could be responsible for Omp85*_Ngo_* recognition by mitochondria. The import signal of Omp85*_Ngo_* is obviously complex and the secondary structure of the β-barrel part plays an important role in mitochondrial targeting of this protein. Our finding that, whereas the removal of the C-terminal phenylalanine alone yields a protein that remains cytosolic, removal of two C-terminal amino acids partially recovers mitochondrial targeting of Omp85*_Ngo_* (Figure 4) speaks in favour of this. We presume that the removal of two amino acids from the C-terminus leads partly to the re-establishment of the secondary structure that is recognized by mitochondria. Further removal of three and more C-terminal amino acids, however, completely impedes mitochondrial targeting (Figure 4).

Similar observations could be made for PorB*_Ngo_*. Again we observe the importance of the C-terminus for mitochondrial targeting, but no precise sequence or region necessary for this process could be pinpointed. We obtained several chimerical proteins made of parts of PorB*_Ngo_* and OmpC*_Eco_* that were not 100% cytosolic or mitochondrial (Figure 6). There probably does not exist only one short import sequence that targets PorB*_Ngo_* to mitochondria, but it is rather the interaction of different parts of this protein and the secondary structure that play a crucial role. In addition, we could show that in contrast to our findings in Omp85*_Ngo_*, the β-sorting signal of PorB*_Ngo_* is not required for its import, indicating that the mechanisms for mediating mitochondrial import are not conserved between neisserial β-barrel proteins (Figure 7).

Omp85*_Ngo_* is imported into human mitochondria and forms complexes in the OMM, whereas PorB*_Ngo_* does not assemble into the OMM upon entering mitochondria [15]. We presumed that this might be due to the differences in the β-sorting signal. However, simple exchange of the β-sorting signal of PorB*_Ngo_* for the one of Omp85*_Ngo_* could not mediate OMM integration of PorB*_Ngo_*, as mitochondria still fragmented and lost their Δψ upon expression of this construct in HeLa cells (Figure 7). Additionally, we observed that, when four or more POTRA domains were removed from Omp85*_Ngo_*, such shortened proteins also failed to integrate into the OMM (Figure 5). This demonstrates that β-sorting signals alone are not sufficient for mediating OMM integration of β-barrel proteins and is in agreement with our previous results, which showed that OMM integration of the mitochondrial porin VDAC depended on single amino acids outside of the β-sorting signal [12].

All our experiments confirmed the previous observation that PorB*_Ngo_* is not integrated into the OMM but rather accumulated in the IMS/IMM [15,16]. In all confocal microscopy experiments, overexpression of PorB*_Ngo_* and all its mutants which were imported into mitochondria resulted in the loss of Δψ, whereas overexpression of Omp85*_Ngo_* and most of its mutants was not deleterious to mitochondria. Our Western blot data showed that in the absence of the functional Omp85*_Ngo_* no PorB*_Ngo_* complexes could be detected in mitochondria (Figure 5C). This finding is in contrast to a study by Jiang and colleagues who observed neisserial PorB*_Ngo_* import into mitochondria and OMM integration in mouse liver cells and concluded that PorB assembly takes place in the OMMs of all eukaryotic cells [22]. If this could be applied to human mitochondria, we would expect to be able to detect at least minor amounts of PorB complexes in mitochondria; however, these complexes are detected only when neisserial Omp85 is present (Figure 5C).

Omp85*_Ngo_* naturally functions in the assembly of β-barrel proteins into the outer membrane of *Neisseria*, together with several accessory lipoproteins [23]. A study by Bos and colleagues demonstrated that in *N. meningitidis* only one POTRA domain of Omp85 is required for its function [6]. In contrast to this finding, we could show here that in human mitochondria at least two POTRA domains are necessary for Omp85*_Ngo_* membrane integration and function and even three POTRA domains seem to be required for oligomerization. The different number of POTRA domains required in bacteria and human mitochondria for Omp85 membrane integration might be explained by the fact that the bacterial accessory lipoproteins normally present together with Omp85*_Ngo_* have no counterparts in human mitochondria, and therefore more POTRA domains might be necessary for the correct assembly of this protein. Likewise, Omp85 might be better adapted to the membrane environment of the bacterial than of the mitochondrial outer membrane. The fact that Omp85-POTRA3 is most probably integrated into the OMM and can assemble PorB but does not form oligomers indicates that Omp85 is also functional as a monomer. This corresponds to the results of Bos et al. who showed that in *N. meningitidis* Omp85 lacking four POTRA domains can mediate β-barrel integration, though probably not forming any oligomers [6]. Our findings furthermore show that the presence of the β-sorting signal of Omp85*_Ngo_* is not sufficient for mediating its OMM integration, but that the POTRA domains also play an important role in this process. Interestingly, when exchanging the N-terminal half of Omp85*_Ngo_*, which contains all POTRA domains, with that of BamA*_Eco_*, the chimerical protein is integrated into the OMM (Figure 2). This suggests that the POTRA domains are sufficiently conserved between these two homologous proteins for mediating OMM integration of Omp85*_Ngo_* by the POTRA domains of BamA*_Eco_*.

In conclusion, our study provides an interesting insight into the complex process of β-barrel protein targeting to mitochondria and OMM integration. However, many questions about the exact nature of the signals and the process of membrane integration remain open, leaving a wide field for future research.
